# Regulation Mechanism of Plant Pigments Biosynthesis: Anthocyanins, Carotenoids, and Betalains

**DOI:** 10.3390/metabo12090871

**Published:** 2022-09-16

**Authors:** Xuecheng Zhao, Yueran Zhang, Tuan Long, Shouchuang Wang, Jun Yang

**Affiliations:** 1 Hainan Yazhou Bay Seed Laboratory, Sanya Nanfan Research Institute of Hainan University, Sanya 572025, China; 2 College of Tropical Crops, Hainan University, Haikou 570228, China

**Keywords:** anthocyanin, carotenoid, betalain, secondary metabolism, transcription factors, regulatory network

## Abstract

Anthocyanins, carotenoids, and betalains are known as the three major pigments in the plant kingdom. Anthocyanins are flavonoids derived from the phenylpropanoid pathway. They undergo acylation and glycosylation in the cytoplasm to produce anthocyanin derivatives and deposits in the cytoplasm. Anthocyanin biosynthesis is regulated by the MBW (comprised by R2R3-MYB, basic helix-loop-helix (bHLH) and WD40) complex. Carotenoids are fat-soluble terpenoids whose synthetic genes also are regulated by the MBW complex. As precursors for the synthesis of hormones and nutrients, carotenoids are not only synthesized in plants, but also synthesized in some fungi and bacteria, and play an important role in photosynthesis. Betalains are special water-soluble pigments that exist only in *Caryophyllaceae* plants. Compared to anthocyanins and carotenoids, the synthesis and regulation mechanism of betalains is simpler, starting from tyrosine, and is only regulated by MYB (myeloblastosis). Recently, a considerable amount of novel information has been gathered on the regulation of plant pigment biosynthesis, specifically with respect to aspects. In this review, we summarize the knowledge and current gaps in our understanding with a view of highlighting opportunities for the development of pigment-rich plants.

## 1. Anthocyanins

**Figure 1:**
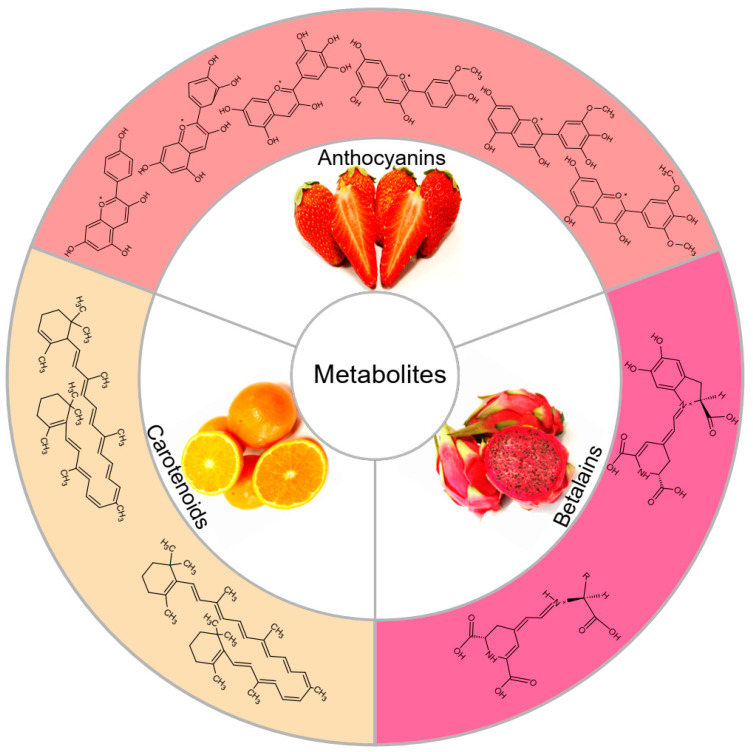
Dietary sources of plant pigments. *Strawberry*, *Citrus sinensis*, and *Pitaya*. Chemical structures of anthocyanins, *α*-Carotene, *β*-Carotene, betaxanthin, and betacyanin.

Anthocyanins are the largest water-soluble pigment, which are mainly found in fruits and vegetables [1] (Figure 1) and are among the terminal products of the flavonoid metabolic pathway [2]. They can be used as visual signals to attract pollinators and seed dispersants. They can not only quench reactive oxygen species as antioxidants, but also absorb ultraviolet and strong light [3,4]. Anthocyanins are mainly present in the form of glycosylation or acylation, and the six main anthocyanidins, cyanidin (Cy), delphinidin (Dp), pelargonidin (Pg), peonidin (Pn), petunidin (Pt), and malvidin (Mv), are generally abundant in plants [1] (Figure 1).

## 2. Biosynthesis of Anthocyanins

**Figure 2:**
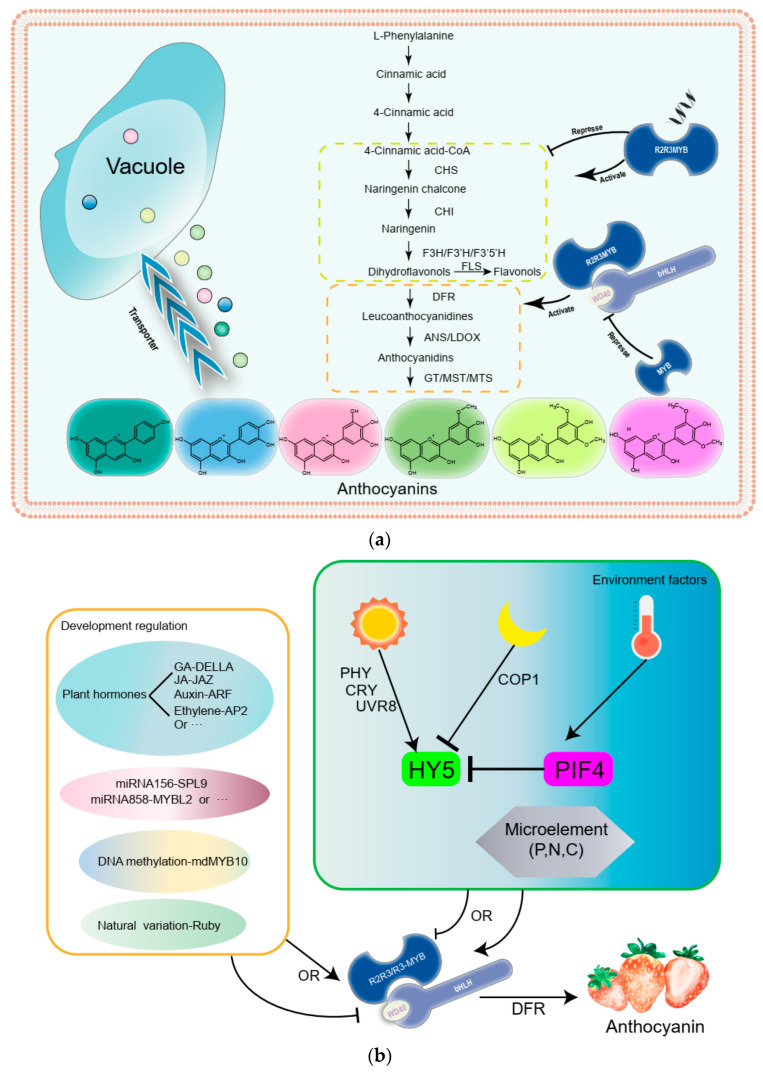
A simplified model of anthocyanin biosynthesis in plants. (**a**) A simplified schematic of the anthocyanin biosynthetic pathway. (**b**) The transcription of anthocyanin biosynthesis genes is regulated by MBW complexes, in which different MYBs enable activate specific parts of the pathway and differentially respond to developmental and environmental cues. MBW: MYB–bHLH–WD40; PAL: phenylalanine ammonia lyase; C4H: cinnamate 4-hydroxylase; 4CL: 4-coumarate-CoA ligase; CHS: chalcone synthase; CHI: chalcone isomerase; F3H: flavanone 3-hydroxylase; F3′H: flavonoid 3′-hydroxylase; F3′5′H: flavonoid 3′5′-hydroxylase; FLS: flavonol synthase; DFR: dihydroflavonol-4-reductase; ANS: anthocyanidin synthase; GA: gibberellin; JA: jasmonate; JAZ: jasmonate ZIM-domain; ARF: auxin response factors; COP1: ubiquitin E3 ligase CONSTITUTIVE PHOTOMORPHOGENIC1; HY5: ELONGATED HYPOCOTYL5; PIF4: PHYTOCHROME INTERACTING FACTOR4; CRY: cryptochrome; PHY: phytochrome (A, B); UVR8: UV RESISTANCE LOCUS; miRNAs: microRNAs.

Anthocyanins are synthesized in the cytosol and then glycosylated and acylated to form various anthocyanin derivatives, which are deposited in vacuoles [5,6]. The flavonoid metabolic pathway is also widely studied, with the core enzymes, such as chalcone synthase (CHS), chalcone isomerase (CHI), flavanone 3-hydroxylase (F3H), and flavonol synthase (FLS), etc., being classified as early biosynthesis genes (EBGs) and dihydroflavonol-4-reductase (DFR), anthocyanin synthetase (ANS), uridine diphosphate-glucose: flavonoid 3-O-glucosyltransferas (UFGT), and glutathione S-transferase (GST) being classified as late biosynthesis genes (LBGs) [7]. DFR plays a crucial role in the biosynthesis of anthocyanins. DFR converts DHK, DHQ, or DHM (dihydrokaempferol, dihydroquercetin, dihydromyricetin) into leucoanthocyanidins, which are then converted into colored anthocyanidins by ANS (same as leucocyanidin oxygenase: LDOX). In addition, in some plant species, such as *Camellia sinensis* and *Petunia*, DFR has strict substrate specificity; this result leads to some species lacking pelargonidin-based anthocyanins [8,9]. Next, anthocyanidins are further decorated by methylation, acylation, and glycosylation [10,11]. Finally, anthocyanins associate with GST and MATE transporters for efficient sequestration into the vacuole (Figure 2a) [12,13]. Usually, both the late biosynthesis genes and transporters embedded in vacuoles are regulated by three transcription factors that form the so-called MBW ternary complexes (containing R2R3-MYB, bHLH, and WD40) (Figure 2a) [6,13,14,15,16,17].

## 3. Transcriptional Regulation of the Anthocyanin Biosynthesis

In many plant species it has been confirmed that anthocyanin biosynthesis is regulated by MYB (R2R3-MYB or R3-MYB) transcription factors at the transcriptional level, such as VvMYB5b in grapes, MdMYB1 in apples, AtPAP1 in *Arabidopsis*, CsMYB75 in *tea*, etc. [18,19,20,21]. These transcription factors promote anthocyanin accumulation by regulating anthocyanin biosynthesis genes, such as *DFR* and *ANS* in the form of the MBW complex. In addition, the MBW complex enables the direct activation of structural genes, such as *ANS*. The MBW complex also activates its own components, such as TT8 and TTG1, as well as the downstream TFs, such as GL3, GL2, TTG2, and usually R2R3- or R3-MYB repressors, such as AtMYBL2 and AtCPC [13,22,23]. R2R3-MYB repressors containing a truncated R2 domain have been shown to be regulators of anthocyanin biosynthesis in plants, such as AtMYBL2, MtMYB2, and PpMYB18 [24,25,26]. These activators and repressors are specifically responsive to certain environmental cues by forming regulatory networks to finetune anthocyanin biosynthesis in plants (Figure 2a) [27]. 

## 4. Developmental Regulation of Anthocyanin Biosynthesis

Anthocyanin biosynthesis is regulated by many hormones, such as IAA and JA. Overexpressing MdIAA26 could promote anthocyanin biosynthesis by upregulating the genes of anthocyanin synthesis in apples [28]. In *Arabidopsis*, the AtJAZ1 protein can interact with AtTT8, AtGL3, and EGL3, interfering with the activity of the MBW complex and resulting in a failure to activate downstream AtDFR, thereby reducing anthocyanin biosynthesis [29]. The AtDELLA1 protein can interact with AtMYBL2 and AtJAZ1, and the interaction between DELLAs and MYBL2/JAZs results in the release of bHLH or MYB subunits and the formation of active MBW complexes, thereby promoting anthocyanin biosynthesis [30]. There is increasing evidence that plant miRNAs play a crucial role in the regulation anthocyanin biosynthesis. For example, the miR156-SPL9 module regulates anthocyanin biosynthesis [31,32,33]. Recently, an exogenous application of non-mature miRNA-encoded miPEP164c was found to be able to stimulate anthocyanin accumulation [34]. In addition, DNA methylation also affects anthocyanin biosynthesis in apples, such as MdMYB10 and MdMYB1 [35,36]. In addition, in *Citrus* species and domesticated cultivars, *Ruby* was found to be an MYB transcription factor controlling anthocyanin biosynthesis. Lacking the functional alleles of *Ruby* led to the loss of the ability to produce anthocyanin in white mutant lemons [37]. In addition, *Noemi* was found to be a bHLH transcription factor and an important determinant of natural variation in flavonoid pigments in citrus [38].

## 5. Light and Temperature Regulation Anthocyanin Biosynthesis

Light and temperature are the main environmental cues affecting anthocyanin synthesis. A bZIP transcription factor ELONGATED HYPOCOTYL5 (HY5) is among the central modulators of light signal coordination through the regulation of appropriate gene expression [39]. HY5 promotes anthocyanin accumulation by activating biosynthetic genes, such as PAP1, Box protein, WRKY72, and MYB10, in different plants [40,41,42,43]. In addition, low or high temperatures can affect anthocyanin accumulation [44,45,46]. Microelements also affect anthocyanin biosynthesis [47,48]. The above results suggest that anthocyanin is not only regulated by the MBW complex but also by developmental and environmental cues (Figure 2b). 

## 6. Carotenoids

Carotenoids are one class of the most important plant pigments, which mainly present yellow, orange, and red colors [49], and there are two main carotenoids, namely α-carotene and β-carotene (Figure 1). Carotenoids are subclass of terpenoids, are lipid-soluble, and are synthesized in chloroplasts, playing a crucial role in the photosynthetic apparatus [7,50,51]. In addition, carotenoids are the synthetic precursors of hormones and nutrition substance, such as ABA and vitamin A [52,53]. In addition to plants, carotenoids also enable biosynthesis in some fungi and bacteria, sometimes in response to different environmental conditions [54]. Carotenoids differ from other pigments in that they play crucial roles in photosynthesis [55]. 

## 7. Carotenoid Biosynthesis Pathway

**Figure 3:**
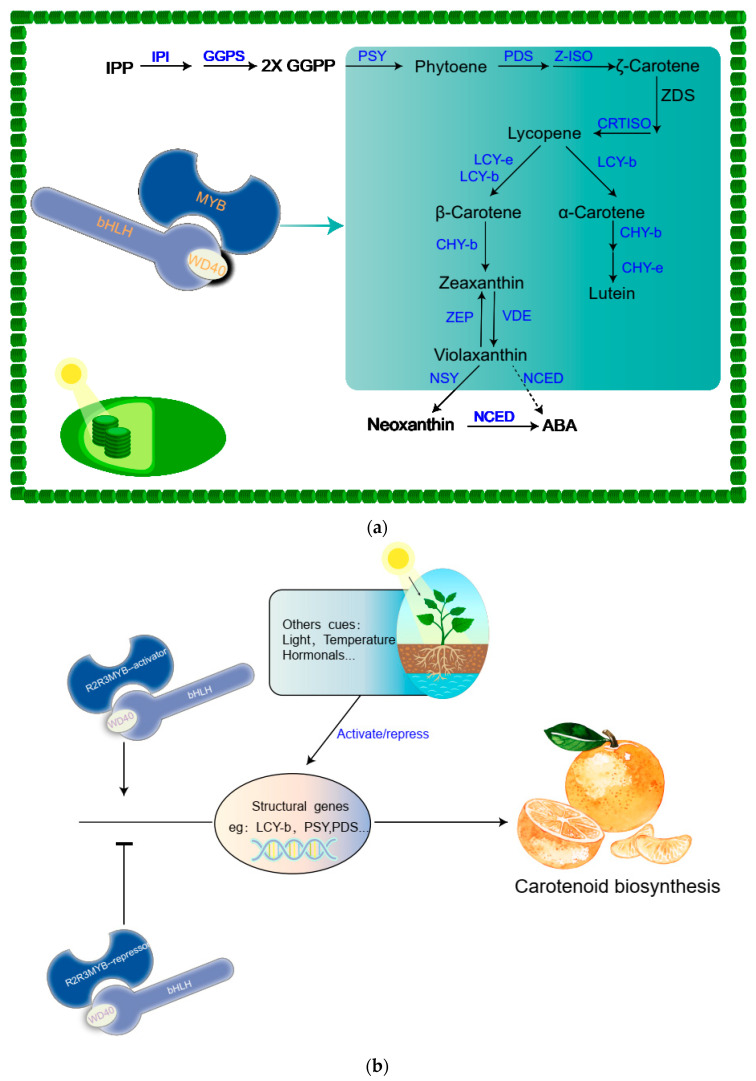
Carotenoid biosynthesis pathway. (**a**) Carotenoid biosynthesis pathway in plants. (**b**) The transcription of carotenoid biosynthesis genes is regulated by MYB complexes, in which different MYBs can activate specific parts of the pathway and respond differentially to developmental and environmental cues. MBW: MYB–bHLH–WD40; IPP: isopentenyl diphosphate; GGPS: geranylgeranyl diphosphate synthase; GGPP: geranylgeranyl diphosphate; IPI: isopentenyl pyrophosphate isomerase; GGDP: geranylgeranyl diphosphate synthase; PSY: phytoene synthase; PDS: phytoene desaturase; ZISO: ζ-carotene isomerase; ZDS: ζ-carotene desaturase; LCYB: lycopene b-cyclase; LCYE: lycopene-cyclase; CHYB: b-ring hydroxylase; CHYE: -ring hydroxylase; ZEP: zeaxanthin epoxidase; VDE: violaxanthin de-epoxidase; CRTISO: carotenoid isomerase; NSY: neoxanthin synthase; NCED: 9-cis-epoxycarotenoid dioxygenase.

For plants, we summarize the carotenoid biosynthesis pathway in Figure 3a. Unlike anthocyanins, carotenoids are synthesized and accumulated in plastids, starting from isopentenyl pyrophosphate (IPP) [56], which suggests that all the carotenoid biosynthetic enzymes are located the plastid [57]. Phytoene synthase (PSY) catalyzes the two-geranylgeranyl diphosphate (GGPP) molecule, forming phytoene (C40), and phytoene desaturase (PDS) and ζ-carotene isomerase (Z-ISO) catalyze the phytoene, forming ζ-carotene. ζ-carotene is then subjected to isomerization reactions catalyzed by ζ-carotene desaturase (ZDS) and carotenoid isomerase (CRTISO), producing lycopene [57]. Lycopene plays a significant role in the metabolic pathway of carotenoids in that lycopene is a central branch point in the carotenoid biosynthetic pathway, which enables the formation α-carotene or β-carotene by cyclization with LYC-b or with LYC-e and LYC-b (Figure 3a). LYC-b and LYC-e governs the bifurcation point of these two types of lycopene cyclization, such as Sl-LYCb2, which can promote β-carotene accumulation in the fruit of beta in tomato. 

Finally, α-carotene forms yellow lutein by a series of hydroxylase reactions with CHY-b and CHY-e. However, β-carotene is then subjected to a series of hydroxylase and de-epoxidase, forming violaxanthin, zeaxanthin, and ABA by zeaxanthin epoxidase (ZEP), violaxanthin de-epoxidase (VDE), and neoxanthin synthase (NXS). Xanthophylls and carotenes enable the generation of different types of carotenoids by further modification (Figure 3a) [58,59]. PSY plays an important role in the carotenoid biosynthesis pathway. A loss-of-function mutant of the Slpsy1 gene presents the yellow-flesh phenotype of fruits in tomatoes [60], which implies that SlPSY was a key control point during carotenoid biosynthesis in tomato fruits. In addition, SlPSY enables the catalyzation of two- GGPP molecules, forming phytoene, which is regarded as the major bottleneck in carbon flux to carotenoids [61].

## 8. Transcriptional Regulation of the Carotenoid Biosynthesis

Carotenoids like anthocyanins are regulated by transcription factors. CsMADS6 can directly activate LCYb1, PSY, and PDS expression, and increase carotenoid accumulation in *Citrus sinensis* [62]. In *Papaya*, CpbHLH1 and CpbHLH2 enable the activation of LCYb1 and PSY expression and promote carotenoid accumulation [63]. MYB7 regulates carotenoid accumulation via the activation of the lycopene beta-cyclase (LCYB) gene and modulates chlorophyll biosynthesis in *Kiwifruit* [64]. SlMYB72 negatively affects chlorophyll and flavonoid accumulation but promotes carotenoid biosynthesis [65]. In addition to the above active transcription factors, there are other types of repressed transcription factors in plants. For example, CrMYB68 enables the repression of the biosynthesis of carotenoids by reducing the expression of CrBCH2 and CrNCED5 in Citrus reticulate [66]. The above studies indicate that there may be a different type of transcription factor enabling the regulation of carotenoid biosynthesis in different plant species (Figure 3b).

In the process of carotenoid regulation, there are two interesting transcription factors, WHITE PETAL1 (WP1) and REDUCED CAROTENOID PIGMENTATION 1 (RCP1). These two transcription factors link anthocyanins to carotenoids, but the regulation mechanism is the opposite. In *M. truncatula* petals, WP1 enables the upregulation of the expression of carotenoid biosynthetic genes, such as MtLYC-e and MtLYC-b, and WP1 could also activate MtCHS and MtANS expression promote anthocyanin accumulation [67]. In addition, WP1 physically interacts with MtTT8 and MtWD40-1. Those results confirm that the conserved MBW complex enables regulatory carotenoid and anthocyanin biosynthesis by activating their biosynthetic genes [67]. RCP1 was also found to be a R2R3-MYB transcription factor, promoting carotenoid accumulation during flower development in *M. lewisii* [68]. Reduction of RCP1 expression led to the downregulation of LCYb1, PSY, and PDS expression and reduced carotenoid accumulation [68]. By contrast, overexpressing RCP1 decreases anthocyanin synthesis; expression of DFR and ANS was repressed [68]. We can observe that WP1 and RCP1 have a similar function to AtPAP1 or AtMYBL2, in that they affect anthocyanin accumulation, which also enables the regulation of carotenoid biosynthesis by the MBW complex. In addition, the above results also show that plants have an efficient mechanism used to regulate plant pigment biosynthesis [67].

## 9. Others Cues That Regulate Carotenoid Biosynthesis

Environmental factors also affect carotenoid biosynthesis, such as shading repression of key carotenoid biosynthetic gene expression, leading to reduced carotenoid accumulate [69]. In *Arabidopsis thaliana*, PIF1 and HY5 antagonistically regulate carotenoid biosynthesis by affecting the expression of PSY and PDS under light conditions [70,71,72]. In addition, low temperatures affect β-carotene content accumulation [73]. Carotenoid biosynthesis is also subject to hormonal regulation. For example, ethylene promotes carotenoid biosynthesis by activating the expression of PSY [74,75]. In addition, Cruz et al. reported an intricate crosstalk between light, ethylene, and auxin signaling that regulates carotenoid biosynthesis [76].

## 10. Betalains

A betalain is a special kind of water-soluble pigment that only exists in *Caryophyllaceae*, such as *Pitaya* (Figure 1). Betalain pigments replace anthocyanins with unrelated red or yellow in *Caryophyllaceae* [77]. Although the colors of betalains and anthocyanins are similar, the synthetic pathways are different. Anthocyanins are phenylalanine-derived, while the synthesis of betalains starts with lysine, and betalains contain the chromophore betalamic acid [77]. In addition, betalains are classified into red betacyanins and yellow betaxanthins, both of which are synthesized in the cytosol and then transported to vacuoles (Figure 1) [78]. Betalains are functionally like anthocyanins, which enable the attraction of animal pollinators and possess high antioxidant and free radical scavenging activities [79,80]. Additionally, betalains are also used as commercial food colorants and additives [81].

## 11. Betalain Biosynthesis Pathway

**Figure 4:**
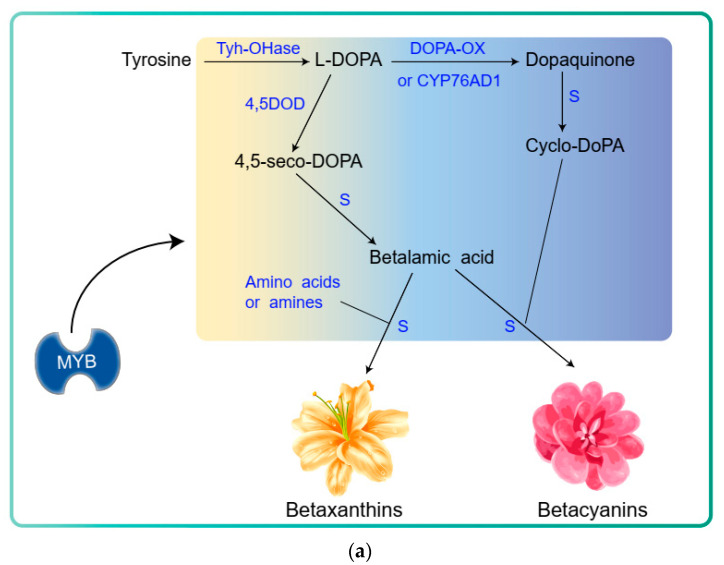

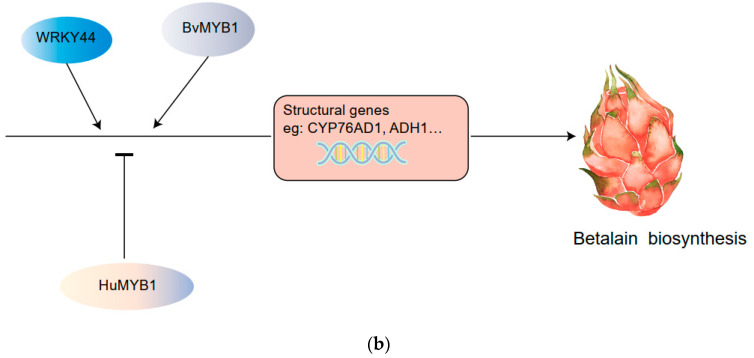
Betalain biosynthesis pathway. (**a**) Betalain biosynthesis pathway in plants. (**b**) The transcription of structural betalain biosynthesis genes is regulated by MYB or other TFs. 4,5DOD: DOPA-4,5-dioxygenase; DOPA-OX: DOPA oxidase; S: spontaneous conversion; Tyh-OHase: tyrosine hydroxylase.

Betalains are derived from the shikimate pathway and synthesized in the cytoplasm and endoplasmic reticulum. Just as with anthocyanins, betalains are eventually stored in the vacuole as glycosides [82,83]. For plants, we summarize the betalain biosynthesis pathway in Figure 4a. Compared to anthocyanins and carotenoids, the synthetic pathway of betalains is simpler in that they only need three main enzymatic catalysts: tyrosine hydroxylation (Tyh-OHase), DOPA oxidase (DOPA-OX), and DOPA-4,5-dioxygenase (4,5DOD) [84]. In the first step, cytochrome P450 enzymes (Tyh-OHase) catalyze tyrosine to form L-DOPA, and L-DOPA is catalyzed by DOPA-OX to form dopaquinone, which it cyclizes to form cyclo-DOPA. In addition, L-DOPA is catalyzed by 4,5-dioxygenase (DODA) to form 4,5-seco-DOPA, then it cyclizes to form to formation betalamic acid [84]. Betalamic acid is a crucial intermediate in the betalain metabolic pathway in that it can spontaneously conjugate with the amino group of the cyclo-DOPA formation of red-violet betacyanins [85], or it spontaneously condenses with the amino or amino group of the amino acid formation of yellow betaxanthins (Figure 4a).

L-DOPA oxidase (CYP76ADs) play a crucial role in the betalain biosynthesis pathway in that it affects the biosynthesis of betacyanins. BvCYP76AD1 not only oxidizes L-DOPA but it also catalyzes the conversion of tyrosine to L-DOPA [86]. However, BvCYP76AD5 and BvCYP76AD6 only exhibit tyrosine hydroxylase activity in Beta vulgaris [87]. Evolutionary analysis results showed that BvCYP76AD1 possess both tyrosine hydroxylase and L-DOPA oxidase activity, falling into the CYP76ADα lineage but BvCYP76AD5 and BvCYP76AD6, which possess only tyrosine hydroxylase activity, belong to the *β* clade [87,88]. 

## 12. Transcriptional Regulation of the Betalain Biosynthesis

In some *Caryophyllales*, a small number of transcription factors were reported to enable the regulation of betalain biosynthesis. In *pitaya*, HuMYB1 is a MYB repressor of betalain biosynthesis, and HmoWRKY44 promotes betalain accumulation by transcriptionally activating the expression of HmoCYP76AD1 [89,90]. A MYB-family TF BvMYB1 is a R2R3-MYB TF, which enables the regulation of betalain biosynthesis [91]. The overexpression of BvMYB1 can promote betalain accumulation by activating BvDODA1 and BvCYP76AD1 expression and silencing BvMYB1, which downregulates betalain biosynthetic genes and reduces pigmentation accumulation in white beets [91]. However, unlike anthocyanin (PAP1-Like) or carotenoid (WP1) MYBs, BvMYB1 lacks conserved amino acids, leading to it not interacting with bHLH members of heterologous MBW complexes [91,92]. However, BvMYB1 enables interaction with bHLH partners when these missing bHLH interaction residues are resurrected [91]. Analysis of phylogenetics indicates that BvMYB1, AtPAP1, and AtMYB114 all belong to the subgroup 6 clade [84]. However, BvMYB1 does not regulate anthocyanin biosynthesis [91]. The above results confirm that BvMYB1 lacks conserved amino acids, could have been deprived of the ability to regulate anthocyanins, and contributed to transcriptional regulation of betalain genes (Figure 4b) [84]. 

## 13. How to Interpret Mutual Exclusion of Betalains and Anthocyanins?

At present, there are three viewpoints on the mutual exclusion of anthocyanins and betalains. Regarding the dual functions of deregulated arena dehydrogenase (ADH), one viewpoint considers that ADH mainly regulates tyrosine synthesis in plants [93] and the overexpression of the *Caryophyllales*-specific deregulated ADH in N. benthamiana results in increased levels of tyrosine as well as depleted levels of phenylalanine [94]. This result led to the fundamental imbalance between tyrosine and phenylalanine-derived pathways in *Caryophyllales*. The second viewpoint considers that BvMYB1 does not interact with bHLH partners derived from anthocyanic model organisms [91]. The third viewpoint considers that the expression difference between DFR and ANS led to the mutual exclusion of anthocyanins and betalains [95,96]. In addition, Polturak et al. [97]. suggested that the truncation of MjANS is another mechanism that may explain the mutual exclusion of anthocyanins and betalains more broadly. 

For the above viewpoints, although each conclusion has sufficient evidence, each viewpoint only represents a specific species, in other words, these conclusions are all limited. Therefore, in order to better understand the mutual exclusion of anthocyanins and betalains, we can further explore these viewpoints so that we can better understand the mutual exclusion mechanism.

## 14. Metabolic Engineering of Pigment Content in Plants or Microbes

At present, the extraction technology of plant pigments is becoming more and more mature. Plant pigments are widely used in the food, cosmetic, and healthcare industries. As we all know, plant pigments of fruits and vegetables, especially anthocyanins, carotenoids, and betalains, play a very important role in human health. We understand the synthetic pathways and regulatory genes of different pigments, which makes it possible to solve the problem of pigment content using the above analysis method. Butelli et al. created the purple tomato using the transgenic approach [98]. Nowadays, although the transgenic approach can improve the yield of pigments, it has always been controversial. Currently, many genomes of fruits and vegetables have been reported, which makes it easy for us to obtain the sequences of genes regulating pigment synthesis. We can reduce the expression of an anthocyanin repressor (MYBL2, SPL, LBD et al.) through genome editing technology (CRISPR/Cas systems). Although the CRISPR/Cas system is also transgenic, a major advantage is that, after the genome is edited, there are no new gene insertions [99]. In addition, microbial fermentation is also a new potential strategy for improving the content of pigments. For example, recombinant expression of DOD in *E. coli* can produce betalamic acid and methionine-betaxanthin in vitro [100]. The pigments directly extracted from plants have the advantages of high safety, nutritious functions, natural coloring, and color tone, and some have special aromatic odors. However, technical means, such as gene editing and enzyme engineering, still need to be further developed [101,102].

## 15. Concluding Remarks and Future Directions

Pigmentation of plants has a long history of research but there is much work to be done. In this review, researchers discussed three pigments in the plant kingdom: anthocyanins, carotenoids, and betalains [103]. By analyzing the regulatory network of anthocyanins and carotenoids, researchers found that both anthocyanin and carotenoid are regulated by the MBW complex, with anthocyanin and carotenoid in competition in some cases. Betalains and anthocyanins look the same in color but there is a big difference between them in regulation [104]. The above results provide an insight for comparative studies of the biosynthetic regulatory controls of anthocyanins, carotenoids, and betalains. In addition, researchers have also described several different types of development and environment cues that regulate the modal of pigments biosynthesis. Although new breakthroughs have been made in the biological regulation of pigment by development and environmental cues, there are still a lot of interesting questions that we need to study in future work.

1. Anthocyanins and carotenoids are regulated by MBW complexes but betalains are not regulated by MBW complexes. MYB (BvMYB1) does not interact with bHLH. Is this mechanism universal or special in the betalain pathway?
2. Does BvMYB1 interact with an unknown bHLH protein?
3. BvMYB1, AtPAP1, and AtMYB114 all belong to the subgroup 6 clade but BvMYB1 does not regulate anthocyanin biosynthesis. Does this result indicate a specific selection for evolution?
4. WP1 associates with MtTT8, and MtWD40-1 regulates both carotenoid and anthocyanin biosynthesis. However, RCP1 positively regulates carotenoid biosynthesis and decreases anthocyanin production. How should this result be interpreted?

Addressing these research questions will help us to have a deeper understanding of the regulation mode of plant pigments, and these questions will also become of high interest for future breeding work.
